# The Landscape of the Anti-Kinase Activity of the IDH1 Inhibitors

**DOI:** 10.3390/cancers12030536

**Published:** 2020-02-26

**Authors:** Katarzyna Malarz, Jacek Mularski, Marcin Pacholczyk, Robert Musiol

**Affiliations:** 1 August Chełkowski Institute of Physics and Silesian Center for Education and Interdisciplinary Research, University of Silesia in Katowice, 75 Pułku Piechoty 1, 41-500 Chorzów, Poland; 2 Institute of Chemistry, University of Silesia in Katowice, 75 Pułku Piechoty 1A, 41-500 Chorzów, Poland; jacek.mularski@gmail.com; 3 Department of Systems Biology and Engineering, Silesian University of Technology, Akademicka 16, 44-100 Gliwice, Poland; marcin.pacholczyk@polsl.pl

**Keywords:** isocitrate dehydrogenase, tyrosine kinase, IDH1 inhibitor, ABL kinase, leukemia, anticancer activity

## Abstract

Isocitrate dehydrogenases constitute a class of enzymes that are crucial for cellular metabolism. The overexpression or mutation of isocitrate dehydrogenases are often found in leukemias, glioblastomas, lung cancers, and ductal pancreatic cancer among others. Mutation R132H, which changes the functionality of an enzyme to produce mutagenic 2-hydroxyglutarate instead of a normal product, is particularly important in this field. A series of inhibitors were described for these enzymes of which ivosidenib was the first to be approved for treating leukemia and bile duct cancers in 2018. Here, we investigated the polypharmacological landscape of the activity for known sulfamoyl derivatives that are inhibitors, which are selective towards IDH1 R132H. These compounds appeared to be effective inhibitors of several non-receptor kinases at a similar level as imatinib and axitinib. The antiproliferative activity of these compounds against a panel of cancer cells was tested and is explained based on the relative expression levels of the investigated proteins. The multitargeted activity of these compounds makes them valuable agents against a wide range of cancers, regardless of the status of IDH1.

## 1. Introduction

**Figure 1:**
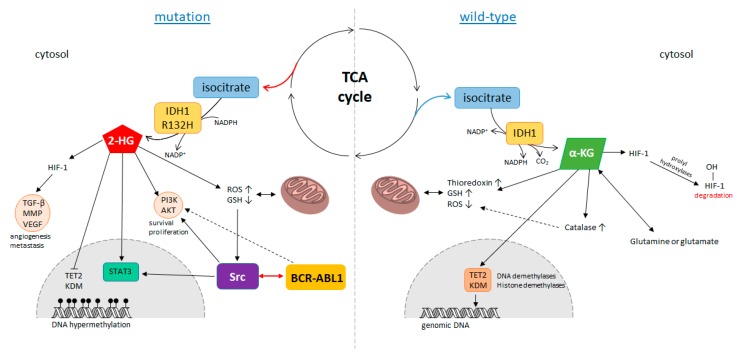
Comparison of the function of the IDH1 enzyme (wild-type) and its mutated form IDH1 R132H.

Isocitrate dehydrogenase 1 (IDH1) is one of the most important enzymes to be involved in cellular metabolism and can be strongly associated with the risk of cancer as well as its clinical outcome. This enzyme catalyses the oxidative decarboxylation of isocitrate into α- ketoglutarate (α-KG) during the tricarboxylic acid cycle (TCA), and utilizes nicotinamide adenine dinucleotide phosphate (NADP^+^) as a cofactor during the reaction, while generating its reduced form: NADPH [1]. IDH1 is localized primarily in the cytosol and peroxisomes, where it plays an important role in maintaining NADPH levels, which are critical for the redox balance that is established by the glutathione and thioredoxin systems [2,3]. An increased level of IDH1 is also linked to catalase activation, which prevents oxidative stress and apoptosis [4,5,6]. However, it is noteworthy that IDH1 also plays an important role in cellular metabolism, proliferation and production energy by participating in the glutamine metabolism and the glucose secretion pathway as well as in the biosynthesis of fatty acids and lipids [7,8]. Cancer cells are characterized by an accelerated metabolism and an increased demand for glucose and other nutrients. Therefore, the overexpression of the wild-type IDH1 is often associated with the uncontrolled growth and survival of numerous cancers, including non-small cell lung carcinoma [9], primary glioblastomas (GBMs) [10], pancreatic ductal adenocarcinoma [11] and several hematological malignancies such as lymphomas or leukemias [12,13]. On the other hand, mutations of the genes encoding the IDH1 enzyme have also been found in several cancers, mainly in about 80% of low-grade gliomas and secondary GBMs, about 40% of T cell lymphomas and in about of 10% of myeloid leukemias [13,14]. The vast majority of IDH1 mutations are heterozygous point mutations that lead to the substitution of arginine in codon 132 within the catalytic pocket of an enzyme. Most of them (over 90%) are related to a missense mutation in a gene, which is based on a single base exchange of guanine to adenine (G395A) that results in the amino acid replacement of arginine by histidine (R132H). Importantly, this change of amino acid is critical for isocitrate binding [15,16]. An IDH1 mutation causes the loss of the normal enzymatic activity of protein, and at the same time allows for the gain of a new function. Therefore, IDH1 R132H is also capable of catalyzing the reduction of α- ketoglutarate to D-2-hydroxyglutarate (2-HG), while converting NADPH to NADP^+^ [17]. An accumulation of the onco-metabolite 2-HG inhibits several 2OG-dependent oxygenases such as the hypoxia-inducible factor (HIF) hydroxylases, histone demethylases (i.e., KDM), 5-methylcytosine hydroxylases (i.e., TET family), and the JmjC domain-containing enzymes, which contributes to epigenetic aberrations and an intensified activity of HIF activity (Figure 1). In turn, the activation of the hypoxic pathways may induce a number of genes (i.e., TGF-β, PDGF, MMPs, VEGF) that affect tumor cell growth and differentiation as well as an invasion, angiogenesis, and metastasis. These activities are usually associated with the promotion of gliomagenesis or leukemogenesis and the malignant progression of brain tumors [17,18,19]. 

**Figure 2:**
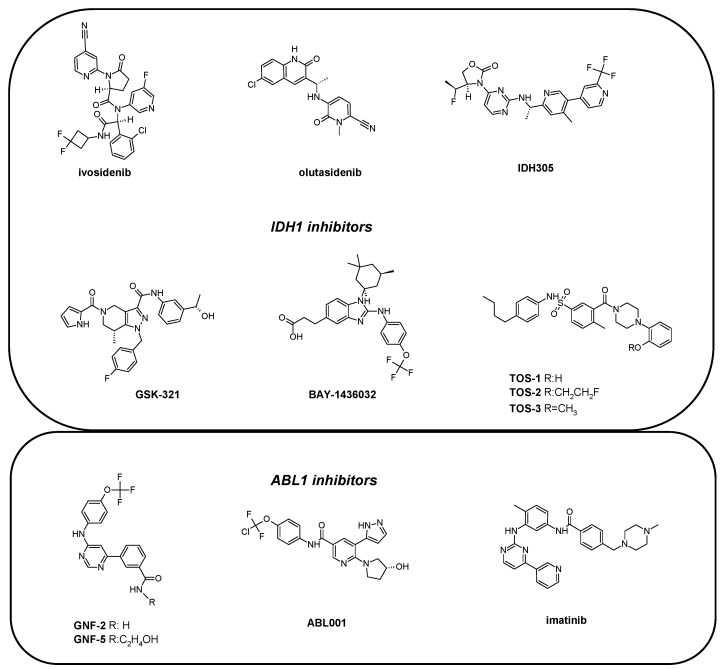
The inhibitors of isocitrate dehydrogenase – IDH1 (upper box) and ABL1 tyrosine kinase (lower box) that are relevant to this study.

Finally, in the middle of 2018, ivosidenib (AG-120), the first IDH1 inhibitor, was introduced to treat leukemia and cholangiocarcinoma [20]. Although it is sometimes described as being R132H selective, it is rather slow binding and non-competitive to the substrate (NADPH) inhibitor of the wild-type IDH1 with activity slightly lower than in the mutant (IC_50_ = 24 vs. 12 nM according to the report [20]). A series of other compounds such as olutasidenib, IDH305 or GSK-321 (see Figure 2) are currently under investigation [21]. The last two are selective inhibitors against the IDH1 mutants, which they bind to an allosteric pocket in order to stabilize an enzyme in a catalytically inactive conformation [22,23]. Their inhibitory activity against IDH1 R132H in biochemical tests were 27 nM and 4.6 nM for IDH305 and GSK-321, respectively. However, in preclinical studies, IDH305 exhibited better pharmacokinetics and efficacy in inhibiting 2-HG production on various xenograft models [23]. All of these facts triggered a strong desire to discover IDH inhibitors that would be suitable for anticancer treatment, which indeed have appeared during the last decade.

An interesting pan-R132 mutant inhibitor with a good selectivity towards the wild-type and low-toxicity BAY1436032 also has good penetrability into brain tissues [24]. This agent has been introduced into clinical trials on a phase I study of AML patients (NCT03127735). More recently butylphenyl sulfonamides (TOS, Figure 2) were described as being selective inhibitors of the R132H mutants [25,26].

Another important molecular target that is widely exploited in the design of drugs suitable for leukemia is proto-oncogenic kinase ABL1. Mutations in this protein, particularly the abnormal gene fusion BCR-ABL called Philadelphia chromosome, are associated with leukemias. The first targeted anticancer drug approved was imatinib, a selective inhibitor of BCR-ABL that has revolutionized cancer therapy [27]. Currently ABL1-oriented drugs are probably the most widely explored group of kinase inhibitors [28]. However contrarily to high expectations imatinib quickly appeared inactive against some specific mutations, specially T315I that means substitution of cytosine to thymine which results in changing at position 315 from threonine to isoleucine [29]. This relatively small shift changes interactions pattern within binding pocket of the enzyme preventing binding first and second generation of inhibitors. More recently, a third generation of ABL1 inhibitors was introduced, e.g., ponatinib, that was approved in 2010 as drug effective against this mutation. Two years later, axitinib was introduced as an inhibitor of VEGF receptors, however it is more promiscuous against other targets including T315I BCR-ABL [30]. 

As presented in Figure 1, BCR-ABL and IDH1 R132H have mutual influence on cell survival and proliferation processes. Considering the significance of these two mutations in developing cancers, exceptionally leukemias, it can be concluded that simultaneously blocking those targets may be an interesting hypothesis for drug design. 

It should be noted here that a highly selective mutant IDH1 inhibitor does not guarantee a good cytotoxic activity in vitro or in vivo. Blocking the 2-HG synthesis itself does not affect the overall metabolism or survival of a cell. Thus, the observed antiproliferative, and even more importantly, the cytotoxic effects are caused by indirect and polypharmacological activities. With this in mind, we decided to perform a more in-depth analysis of the polypharmacological activity of TOS-1 and TOS-2. The structural similarities between them and the inhibitors of ABL1 tyrosine kinase such as imatinib prompted us to evaluate its activity in this area. Interestingly, TOS inhibitors have also shown some similarities to abl001, which is one of allosteric inhibitors of ABL1 [31]. Therefore, in present work we investigated the TOS polypharmacological landscape of activity within the scope of the direct and allosteric inhibition of the non-receptor tyrosine kinases along with their anticancer activity on various cell lines. We decided to exploit a typical approach of testing the activity against a panel of enzymes, including primary screening in one concentration and determination of IC_50_ for active molecules. Anticancer activity was tested in a colorimetric test evaluating the metabolic viability of the cells. Independently we have assayed the expression level for kinases that are targets for the molecules of interest. This approach was useful for a correlation enzymatic assay with real antiproliferative activity towards living cells. Finally, our hypothesis of allosteric inhibition of ABL1 by TOS was evaluated by combination drug assay with calculation of interaction type between competitive and allosteric inhibitor. As a proof of concept, we analyzed in silico interactions between protein and inhibitor and determined a plausible pharmacophore model for them. We believe that our results appeared interesting and confirmed a mutual IDH-ABL activity for those inhibitors. With this in mind, our findings have opened a new and unexplored field of potential dual inhibitors aiming at kinase and glucose metabolic pathways.

## 2. Results

### 2.1. Chemistry

The synthetic procedures for the multistep preparation of the target compounds are available elsewhere [26]. In our work, however, we found it necessary to modify this approach in order to obtain a better yield and to use microwave irradiation.

**Scheme 1:**
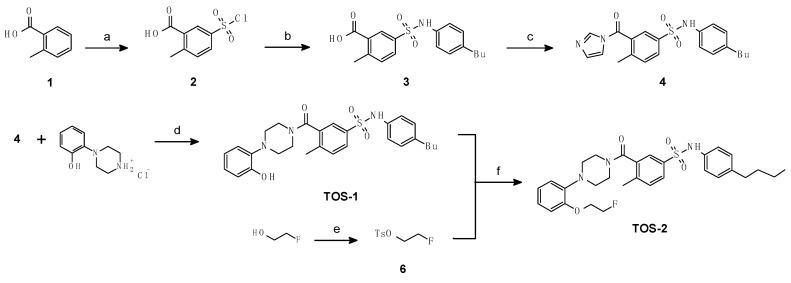
Synthesis of the tested compounds (**a**) HSO_3_Cl, RT, 24 h; (**b**) Bu-C_6_H_4_-NH_2_ ·HCl, ACN/Py (1:1 *v*/*v*), DMAP, (MW: 100 W, 80 °C, 60 min); (**c**) 1,1′-carbonyldiimidazole, CH_2_Cl_2_, RT, 15 min; (**d**) IPA, (MW: 100 W, 80 °C, 60 min); (**e**) TsCl, Et_3_N, RT, 2 h; (**f**) Cs_2_CO_3_, DMF, (MW: 100 W, 120 °C, 15 min).

Commercially available 1 was reacted with chlorosulfonic acid (Scheme 1) to obtain 2, which was then microwaved with p-butylaniline hydrochloride in the presence of pyridine and DMAP. The sulfamoyl derivative 3 was then reacted with 1,1′-carbonyldiimidazole and product 4 without isolation was further irradiated with 2-hydroxyphenlypiperazine hydrochloride in order to obtain TOS-1. Its fluoroethyl derivative TOS-2 was obtained from TOS-1 using microwave irradiation and freshly prepared 6. 

### 2.2. Inhibitory Activity against the Non-Receptor Tyrosine Kinases

The inhibitory potency against a series of tyrosine kinases has primarily been evaluated for two IDH inhibitors. Our panel contained nine non-receptor kinases: the ABL1, BRK, BTK, and Src families of kinases (CSK, Fyn A, Lck, Lyn B, Src). For the ABL1 kinase, we included the native as well as the single-point mutation T315I, that is responsible for resistance against first generations of TKI as discussed above. We have performed all inhibition tests on commercially available enzymes, including the mutant ABL1 T315I. As standards, two commercially available inhibitors, imatinib which is selective towards ABL1 kinase, and axitinib, which is a non-selective highly potent second-generation inhibitor, were used. Noticeably, imatinib is only active against native proteins while axitinib has an even stronger activity against the mutant. For axitinib, the IC_50_ values that were calculated were 0.452 μM for the ABL1 native and 0.075 μM for the mutated T315I. The results are presented in Table 1 as a percentage of blocked enzyme in 0.5 μM of the drug. 

**Figure 3:**
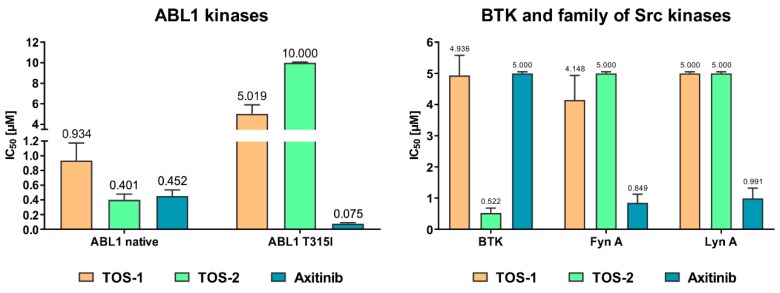
The IC_50_ values of the tested inhibitors against the non-receptor tyrosine kinases.

Both TOS expressed level of activity similar to axitinib and imatinib against ABL1. The T315I mutant was more resistant to all compounds with exception of axitinib. Interestingly TOS-1 and TOS-2 have more complex profile with activities towards other kinases that were tested. In Figure 3, activities that causes a 50% reduction of enzyme activity (IC_50_) are presented for the most active compounds. 

**Figure 4:**
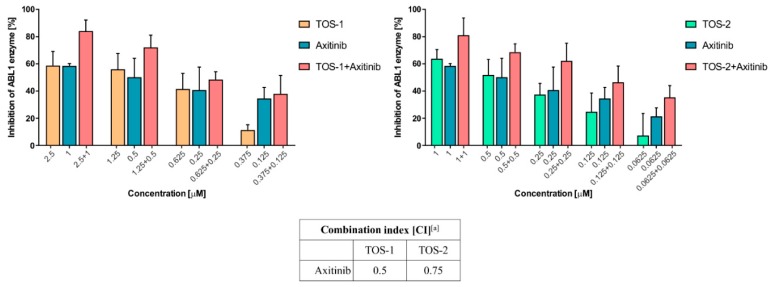
Inhibition of the ABL1 enzyme by TOS-1, TOS-2 and their combination with axitinib. The combination indexes (CI) for the combinations of the TOS derivatives and axitinib were calculated using CompuSyn software; ^[a]^for Fa = 0.9, CI > 1 – antagonism, CI = 1 – additivity, CI < 1 – synergy.

Further, we decided to measure the inhibition effects on the ABL1 enzyme after it had been treated with a combination of the two TOS inhibitors and axitinib. The enzymatic activity after the simultaneous application of those inhibitors was measured using an ADP-Glo luminescence assay, while the concentrations of the tested inhibitors were selected based on their IC_50_ (Figure 3 and Table S1). The types of interactions between the two tested IDH1 inhibitors and axitinib were calculated using the method of Chou and Talay [32,33]. The results of these combination studies are presented in Figure 4. Interestingly, we observed a synergy for both of the tested TOS inhibitors. However, a stronger synergy was detected for the combination of the TOS-1 inhibitor and axitinib (CI value = 0.5). In turn, for the combination of the fluoroethyl derivative and axitinib, we observed a less profound effect and the CI value was 0.75. 

### 2.3. Cytotoxicity Studies

The next step was to characterize the antiproliferative activity of all of the tested inhibitors. For this purpose, six cell lines that differed in their origin were selected, including leukemia (K562), glioblastoma (U-251), colon (HCT 116), breast (MCF-7) and pancreatic (PANC-1) cancers. The activities shown in Table 2 were obtained in MTS test that is focused on metabolic activity of the cells by measuring NAD(P)H-dependent dehydrogenase activity. Both axitinib and imatinib maintain a high activity level against the K562 leukemia cell lines with a very high selectivity. The IC_50_ values that were obtained were 0.10 μM and 0.13 μM, respectively. In addition, apparently within tested concentration, these drugs have no effect on the survival of the other tested tumor cell lines (IC_50_ >30 µM). Both IDH1 inhibitors were considerably less active and less selective against the K562 cell line. The calculated IC_50_ values were 12.65 μM for TOS-1 and 17.26 µM for the fluoroethyl derivative. MCF-7 is the cell line that is the second most vulnerable to the effects of the tested sulfamoyl derivatives. A higher activity was observed for the TOS-2 derivative (IC_50_ = 15.59 µM). Moreover, in case PANC-1 cell line, the TOS-2 was expressed high activity (IC_50_ = 18.93 µM). Both colon cell lines were rather resistant for tested inhibitors, the calculated IC_50_ values were in the range 24.44 to 28.85.

### 2.4. Landscape of the Gene Expression of IDH1 and the Non-Receptor Tyrosine Kinases

**Figure 5:**
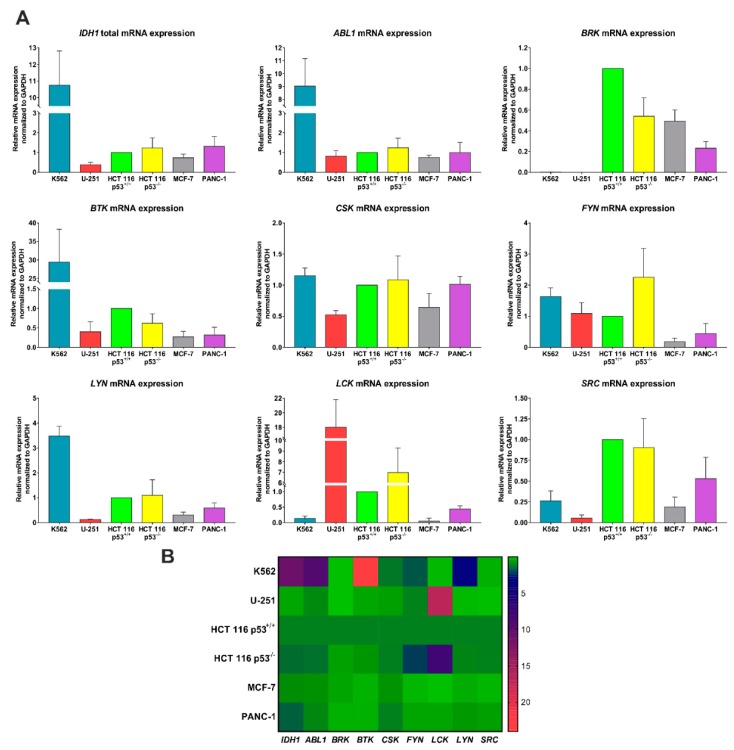
mRNA expression of the *IDH1*, *ABL1*, *BTK*, *BRK* and Src family tyrosine kinases in the various cancer cell lines (K562, U-251, HCT 116 p53^+/+^, HCT 116 p53^-/-^, MCF-7 and PANC-1). The results are shown as the mean ± SD of four independent measurements, each in triplicate. The data were normalized to the HCT 116 p53^+/+^ (one value) (**A**). Heat map displaying the expression of IDH1 and the kinase landscapes in the tested cancer cell lines (**B**).

In order to correlate the cytotoxic activity with the inhibition of the kinases, we measured the expression of the relevant genes. The relative mRNA expression of IDH1 and other kinases in tested cancer cell lines, such as K562, U-251, HCT 116, MCF-7 and PANC-1, we determined using quantitative Real-Time PCR. For each PCR reaction, we used specifically designed primers for selected target genes sequences (See Table S2 in the supporting information). The products obtained in the PCR reaction were confirmed by melting curve analysis. The expression of target genes was normalized to internal control and housekeeping genes according to the method of Livak and Schmittgen [34]. The results, which were normalized to average HCT 116 p53^+/+^ cells, are presented in Figure 5. 

### 2.5. Inhibitory Activity against the Mutant IDH1 Enzyme

**Figure 6:**
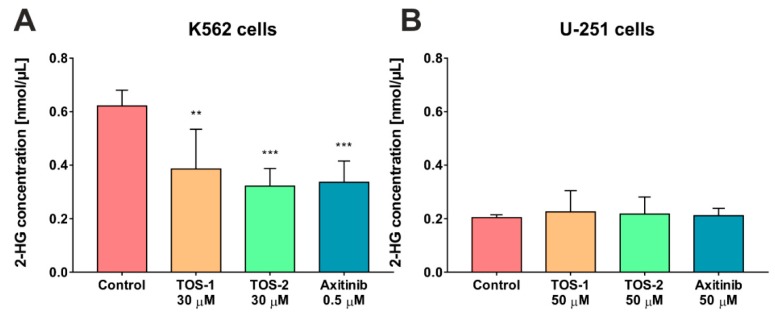
Influence of the tested inhibitors – TOS-1, TOS-2 and axitinib on 2-HG production in the K562 (**A**) and U-251 cells (**B**). Data were analyzed using a one-way ANOVA with Bonferroni’s post-hoc test: ** *p* < 0.01, *** *p* < 0.001 compared to the control (untreated cells).

The interesting differences in the gene expression profile between leukemia and glioblastoma cells prompted us to determine the inhibitory potential of the tested compounds against the mutant IDH1 enzyme. As mentioned above, IDH1 R132H has the ability to convert α-ketoglutarate into 2-hydroxyglutarate (2-HG) in the presence of NADP. Therefore, we decided to analyze the level of 2-HG in the cells after they had been treated with the TOS-1 and TOS-2 derivatives and axitinib. The results of these experiments are presented in Figure 6.

Generally, we observed some differences in the basal level of 2-HG in the tested K562 and U-251 cell lines. In the case of the leukemia cells, we noticed about a three-fold higher basal concentration level of the D-2-HG product compared to the U-251 cells. 

### 2.6. Molecular Docking Studies

**Figure 7:**
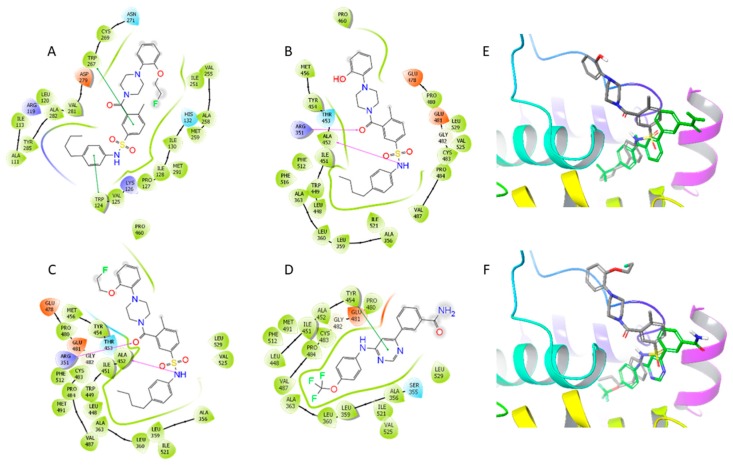
(**A**). TOS-2 docked to IDH1 R132H (PDB ID: 5DE1); (**B**). TOS-1 docked to the allosteric site of ABL1, PDB ID: 3K5V; (**C**). TOS-2 docked to the allosteric site of ABL1, PDB ID: 3K5V; (**D**). GNF-2 in its conformation from cocrystalling [35]; (**E**). and (**F**). TOS-1 and TOS-2, respectively, and GNF-2 in the overlay after docking to 3K5V.

To gain more a detailed insight into the possible activity mode of both TOS on ABL1, we performed some docking studies. The allosteric hypothesis was confirmed in in silico experiments with the available structures of the proteins. We selected the ABL wild type (PDB ID: 4WA9 and 3K5V, which are available with imatinib and GNF-2 docked) and T315I (4TWP) as well as the IDH1 (5DE1) structure. The first results from the docking experiments are presented in Figure 7. Pharmacophore models for both enzymes were generated in Maestro Schrodinger by sampling the respective protein pockets with a set of the fragments that were generated by the decremental fragmentation of structure inhibitors that were investigated (Table S3 and Figure S1).

## 3. Discussion 

According to our expectations, both tosylamides appeared active against ABL1 on the same level as both standard drugs. TOS were less active against T315I and imatinib has lost its potency against this enzyme, while axitinib was even more active than against wild type protein. As was reported by Pemovska et al., the specific binding conformation, which is possible in axitinib and the A-loop position of a mutated enzyme [30]. For axitinib, the IC_50_ values that were calculated were 0.452 μM for the ABL1 native and 0.075 μM for the mutated T315I. Interestingly, TOS-1 and TOS-2 expresses substantially different activity patterns despite their structural similarity. The fluoroethyl derivative appeared more active and selective towards the ABL1 and BTK kinases. The calculated IC_50_ value was 0.401 μM against the ABL1 wild type, while for the mutated form, it was not possible to calculate it within the range of the tested concentrations (>10 μM). For BTK kinase, the obtained IC_50_ value was similar (0.522 μM). By contrast, TOS-1 was the most active against ABL1 (IC_50_ = 0.934 μM). Additionally, it had a moderate activity against BTK, CSK and Lck at a similar level. This derivative also had some effect on BRK and Fyn A. These differences can only be partially explained by the changed interaction profile in those two derivatives. 

As mentioned above, TOS was somewhat similar to the allosteric inhibitors of ABL1. With this in mind, we focused on such a possibility. Allosteric inhibitors such as GNF-2 and abl001 bind to the myristate pocket of ABL1. However, both pockets are separated by a 25 Å, binding of the allosteric inhibitor trigger conformational changes in ATP-binding pocket. Thus, in combination, those inhibitors are known to interact synergistically. The simultaneous binding of both the ATP- and myristate-competitive inhibitors (e.g. axitinib and GNF-2) has been determined to be responsible for the conformational and dynamic changes that might help to overcome resistance [36]. Such combinations are effective in both the mutated and wild-type BCR-ABL enzymes [37]. We decided to measure the inhibition effects on the ABL1 enzyme after it had been treated with a combination of the two TOS inhibitors and axitinib. The enzymatic activity after the simultaneous application of those inhibitors was measured using an ADP-Glo luminescence assay, while the concentrations of the tested inhibitors were selected based on their IC_50_ (Figure 3 and Table S1). The type of interactions between the two tested IDH1 inhibitors and axitinib were calculated using the method of Chou and Talay [32,33]. The results of these combination studies are presented in Figure 4. Interestingly, we observed a synergy for both of the tested TOS inhibitors. However, a stronger synergy was detected for the combination of the TOS-1 inhibitor and axitinib (CI value = 0.5). In turn, for the combination of the fluoroethyl derivative and axitinib, we observed a less profound effect and the CI value was 0.75. 

The antiproliferative activity measured in the cells is always an effect of various interactions between drug and molecules and pathways. To better investigate this aspect of our study, we selected several different cancerous cell lines. Chronic myeloid leukemia (CML) is associated with a chromosomal aberration (Philadelphia chromosome), which involves a translocation between chromosomes 9 and 22 that results in a BCR-ABL1 oncogenic fusion protein [38]. This mutation induces an increased and constitutive activity of the BCR-ABL1 kinase, which may confer the ability to grow in the absence of growth factors, to metastasize and to resist the apoptosis that is caused by a wide range of anticancer drugs on cells [39]. Therefore, the leukemia cell line is a primary choice for ABL1-targeted therapy. Moreover, recently the importance of IDH mutations in leukemias has been stressed and several new drug candidates are currently investigated [22,40,41]. For the colon adenocarcinoma, we also selected mutants with a deletion of the TP53 gene (HCT 116 p53^-/-^). Mutations in this gene are present in more than 50% of all cancers and correspond to resistance, higher metastatic and a worse general prognosis [42,43,44,45]. The p53 protein is responsible for detecting DNA damage and regulating the cell cycle and repair processes: its pathway is driven by specific kinases, including c-ABL, Src, and Aurora kinase [46,47,48]. However, in both the wild-type and p53 null colon cancer cell lines, the c-ABL regulation of the p53 downward proteins were found to be altered [49]. Another common cancer is breast cancer, which is known for the oncogenic receptor kinase HER2, although other kinases also play important roles, including Lyn, which is responsible for a resistance to anti-estrogen therapy [50]. BTK and Src, which are involved in the apoptosis pathway that is induced by anticancer drugs, may also influence the malignancy of breast cancer [51,52]. By contrast, the pancreatic cancers and glioblastomas are the most aggressive cancers and have a generally poor prognosis and short survival time [53]. It is worth noting that the overexpression of IDH1 as well as mutations of this protein are most frequently found in a primary and secondary glioblastoma. However, a recent study demonstrated that a high expression level of *IDH1* was observed in primary and metastatic pancreatic cancers [11]. Interestingly, the malignancy of these cancer types can often be connected with p53 mutations [54]. However, in leukemias, p53 is important and has influences the outcome of kinase-dependent therapy [55,56]. The explanation of the susceptibility of these two cell lines to the tested compounds may be indirectly related to the BTK protein, which is involved in the development of CML and breast cancer. It is also worth noting that both of the IDH1 inhibitors had the ability to inhibit BTK kinase. Interestingly, they were more active against the K562 and MCF-7 lines, whereas the U-251 glioblastoma cells were resistant to most of the compounds that were tested. TOS-1 and TOS-2 were slightly less active against the colon cancer cells with a deletion in TP53 gene, which do not express the p53 protein. The IC_50_ values that were obtained were about 25 µM for both of the tested compounds on the HCT 116 p53^+/+^ cells. A very similar level of activity was observed for TOS-1 in the PANC-1 cell line.

At least partially these results can be explained by different expression of the specific genes. As was expected, both the *IDH* and *ABL* genes were overexpressed in the leukemia cells. Interestingly, the expression levels of the *LYN* and *BTK* genes were also several times higher than in any of the other cells that were tested. This observation is in good agreement with the literature data where the activity of those kinases has been correlated with the malignancy of CML [57,58]. Conversely, the K562 cells had a significantly lower expression level of the *SRC* kinases, particularly the *LCK* and *BRK* genes. Interestingly, *LCK* was found to be significantly increased in the glioblastoma cells U-251. Although the role of various proteins from the Src family has been discussed in the development of brain cancer and the response to therapy, the lymphocyte-specific protein has not yet been elucidated. The literature reports remain inconclusive and suggest both a minor [59] or major [60] role in the growth and therapeutic response of this kinase, among others, of the Src family. However, a recent report that was published by Zepecki et al. suggests that the inhibition of *LCK* may reduce the mobility of glioblastoma cells within the parenchyma, which is one of the reasons for its aggressiveness and metastatic activity [61]. Conspicuously, the glioblastoma cell line had a characteristically low level of the expression all of the genes that were tested except *LCK*. Remarkably, the mRNA level for the *IDH1* transcription was also the lowest among all of the cell lines that were investigated, which correlates well with the low basal level of IDH1 or the IDH1 R132H mutant in those U-251 cells, which was previously reported by Oizel et al. [62] This also explains the relative resistance of glioblastoma cells to both the tosylamides and two other inhibitors. Specifically, TOS-1, which is slightly active against the *LCK* and *CSK* kinases, was more active against U-251 than the other compounds.

Similarly, the observations of reducing the IDH1 activity by TOS and axitinib are in agreement with the qRT-PCR analysis of genes, where we observed a high level of the expression of the total *IDH1* in K562 but not in the U-251 cells (Figure 5A). Moreover, in the case of the K562 cells, we observed a significant decrease in the D-2-HG level to 24% and 48% after a 24-h incubation with the TOS-1 and TOS-2 derivatives, respectively (Figure 6A). This effect confirms the inhibitory potential of these compounds against IDH1 R132H, which Chitneni et al. demonstrated in enzymatic in vitro assays and in astrocytoma cells [26]. Interestingly, the potent multi-kinase inhibitor, axitinib, also reduced the level of the 2-hydroxyglutarate (46%) in K562 cells. However, we did not observe any changes in 2-HG production in the glioblastoma cells after a 24-h incubation with all of the tested compounds (Figure 6B). This effect could be correlated with the very low basal level of the IDH1 expression that was observed in U-251 cells. Those cells were characterized by a higher level of Lck, followed by the Fyn and CSK proteins. Moreover, this supports our hypothesis about the inhibitory effect that is exerted by all of the compounds that were tested including axitinib. 

It is noteworthy that the mutation of the IDH1 enzyme and hypoxia conditions caused an increase in the production of 2-HG in c the ells [63]. More recently, Seok et al. demonstrated that D2-HG induced angiogenic activity through the vascular endothelial growth factor receptor 2 (VEGFR2) signaling pathway and the activation of the MMP2 protein [64]. Thus, the inhibition of those pathways would be particularly beneficial in treating some cancers.

It was also interesting to analyze the interactions of TOS-2 with IDH1 as this was not yet presented elsewhere. The inhibitor molecule interacts with aromatic ring of Trp-124 and Trp-267 and fits the pocket of Tyr-285 in a similar way (Figure 7A). Like the known inhibitor GSK321, which has been reported [22], TOS-2 does not interact with the His-132 residue. One molecule of GSK321 binds to each of the IDH1 R132H monomers in a pocket that is lined on three sides by Ile-128, Pro-127, Trp-124, Arg-119, and Leu-120. The remaining side is formed by residues (e.g., Val-281, Gly-284 and Tyr-285). The presented results were obtained from one chain of the IDH1 R132H homodimer (PDB ID: 5DE1 -chain A). The crystal structure showed a homodimer that was bound with two molecules of GSK321 and there was no direct interaction identified between the ligand and the second chain of the protein. In order to evaluate the myristate pocket binding hypothesis, we analyzed the interaction of both TOS docked into ABL1 (PDB ID: 3K5V) compared to GNF-2 (Figure 7B–F), which is a known, selective allosteric inhibitor of ABL1 that has a similar level of activity (~1 μM [65]) towards recombinant protein [37,66]. Interestingly, although both TOS-1 and TOS-2 fit into the allosteric pocket of ABL1 well, the interactions that were observed differed from those that were present in GNF-2. The TOS inhibitors formed H-bonds with Ala-452 and Arg-351 and penetrated the pocket near the Leu-360-Ala-363 region (Figure 7B,C). Conversely, the GNF-2 built a water-mediated hydrogen bond with Tyr-454 and penetrated the Leu-360-Ala-363 region with the trifluoromethyl group (Figure 7D). Apparently, the long but flexible structure of TOS-1 and TOS-2 enabled a good fit into the allosteric pocket and the formation of strong interactions. However, the fluoroethyl substituent in TOS-2 supposedly does not increase the direct interactions enough to explain why its activity is better than TOS-1. 

**Figure 8:**
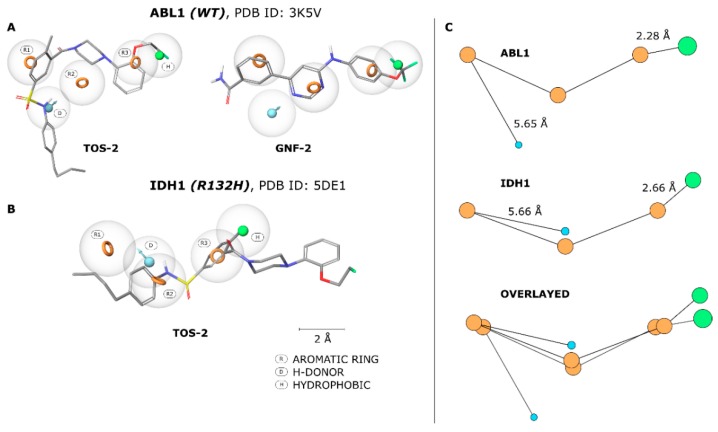
(**A**). TOS-2 and GNF-2 matched into the pharmacophore based on the ABL1 WT myristate binding site. (**B**). TOS-2 matched into the pharmacophore based on the IDH1 R132H allosteric site. The Figure 2. Å. (**C**). Graphic representations of the pharmacophores: the aromatic ring features are aligned to the page plane. The circle size is relative to the plane distance.

These promising results prompted us to build and compare pharmacophore models for the allosteric site of ABL1 and IDH1. The respective docking poses of TOS-2 fit into the pharmacophore hypotheses differently as can be seen in Figure 8A,B, which is in agreement with the results that were presented above (Figure 7). However, its flexible structure is able to cover most of the features that can be revealed at the myristate binding site of ABL1. Moreover, there is a common set of features that can be distinguished in those pharmacophore hypotheses, as is presented in Figure 8C. It is noteworthy that the differences in those hypotheses do not exceed 1 angstrom, which in turn can be eliminated by the normal flexibility of the chemical bonds. Interestingly, there were pharmacophore models that forced slightly different poses of inhibitors to those that were obtained from direct docking (Figure S2). Docking to ABL1 revealed interactions between the hydrogen atom of the sulfonamide moiety and the oxygen atom of Tyr454. This interaction matched the H-donor feature of the pharmacophore model. Moreover, docking revealed an aromatic hydrogen bond between the sulfonamide group and the ortho hydrogen atom of tyrosine. An adjacent diamide aromatic ring matches to R1 pharmacophore feature, while aromatic ring with trifluoromethoxyl substituent matched the R3 aromatic region of the pharmacophore. This feature should be interpreted as a more hydrophobic pocket because no aromatic amino acids residues were identified in its vicinity. This observation is in agreement with the literature reports [67]. The piperazyl moiety of TOS-2 can be considered to be a linker although the analogous pyrimidinyl group in GNF-2 is able to form a water-mediated hydrogen bond with Tyr454. Similarly, the H-donor feature of the pharmacophore that was built on IDH1 was at a nearly identical distance to R1 to the one described earlier, even if it is out of the plane that was defined by three aromatic features. The same fragment of TOS-2 is able to form the necessary interactions with the Ile-128 backbone as in the case of ABL1. Tyr285 interacts with the butylphenyl moiety of an inhibitor while Trp124 interacts with the diamide ring. Additionally, the aromatic hydrogen bond was identified between the oxygen atom of the piperazyl amide and the indole ring of Tep124. The methyl group matched the hydrophobic feature of the model, while Trp267 interacted with the diamide ring. However, it should be noted that the second protein chain of the dimer can add some stabilizing interactions to this model. To summarize, the similarities in both enzymes provided enough evidence to support the hypothesis of a dual inhibitory activity of TOS. At the same time, the differences between the docking poses of TOS-2 in both protein pockets could suggest that this molecule has some features of a fused-type inhibitor, which in turn is particularly worth investigating further. 

## 4. Materials and Methods

The Fourier transform nuclear magnetic resonance spectra of the sample solutions were obtained using a Bruker Ascend 500 for 1H (500 MHz), and the chemical shifts are reported in δ units (parts per million) relative to tetramethylsilane and the residual solvent peaks are used as the reference. The high-resolution mass spectra were measured using a DionexUltiMate® 3000 high-performance liquid chromatograph (Thermo Fisher Scientific, West Palm Beach, FL, USA) coupled with an LTQ Orbitrap XL™ Hybrid Ion Trap-Orbitrap Fourier Transform Mass Spectrometer (Thermo Fisher Scientific) with an injection into HESI II in the positive or negative modes. The chromatographic separations were performed on a Teledyne ISCO Combiflash Rf150+. The melting point was determined on an MPA100 Melting Point Apparatus from Stanford Research Systems. The imatinib mesylate and axitinib were purchased from Sigma-Aldrich.

### 4.1. Synthesis of Compounds

The synthetic procedures for the multistep preparation of the target compounds, along with their characterization, have been published in work of Chitnani et al. [26]. In our work, however, we found it necessary to modify this approach in order to obtain a better yield and to use microwave irradiation. Therefore full experimental procedures are given below. The sequential synthetic approach has been explained in Scheme 1.

#### 4.1.1. 4. -(2-hydroxyphenyl)piperazin-1-ium Chloride

The 2M methanolic hydrochloride was added to a solution of 4-(2-hydroxyphenyl)piperazine in methanol. Then, after 10 minutes, diethyl ether was added until the precipitate was formed. A white solid was collected and washed with acetone. The solid was dried to a constant mass.

#### 4.1.2. 4. -butylalanine Hydrochloride

Briefly, 4-bytulalanine was added to 1 M ethereal hydrochloride. The precipitate was collected and washed with diethyl ether.

#### 4.1.3. 5. -(chlorosulfonyl)-2-methylbenzoic Acid (2)

The product was obtained from *o*-toluic acid (1 on Scheme 1). Product 1 was added (4080 mg, 30 mmol) to 10 mL of well-stirred chlorosulfonic acid in small amounts. The reaction mixture was stirred for 24 h at 20 °C and then added dropwise to 50 mL of stirred water with ice. The precipitated solid was collected and washed twice with water. The compound was dried over phosphorus pentoxide for 24 h to yield 2 (5262 mg, 75%) as a white solid; ^1^H NMR (CDCl_3_, 500 MHz;): δ 8.76 (d, 1H, *J* = 2.2 Hz), 8.13 (dd, 1H, *J* = 8.2, 2.2 Hz), 7.59 (dd, 1H, *J* = 8.3, 0.4 Hz), 2.83 (s, 3H).

#### 4.1.4. 5. -[(4-butylphenyl)sulfamoyl]-2-methylbenzoic Acid (3)

Product 2 (235 mg, 2.5 mmol), p-butylaniline hydrochloride (537 mg, 2.5 mmol) and DMAP (40 mg, 0.33 mmol) was added to a mixture of acetonitrile and pyridine (3 mL, 1:1 *v*/*v*). The reaction mixture was irradiated with microwaves (80 °C, 100 W, 1 h). The reaction mixture was diluted with 100 mL of CH_2_Cl_2_ and washed with a 1N HCl solution, and then with brine. The organic phase was dried over MgSO_4_ and the volatiles were evaporated under reduced pressure. The off-white solid that was obtained was washed with petroleum ether to yield Product 3 (434 mg, 50%) as a white solid. ^1^H NMR ((CD_3_)_2_SO, 500 MHz): δ 13.26 (s, 1H), 10.16 (s, 1H), 8.18 (d, 1H, *J* = 2.0 Hz), 7.73 (dd, 1H, *J* = 8.1, 2.1 Hz), 7.46 (d, 1H, *J* = 8.1 Hz), 7.04 (d, 2H, *J* = 8.4 Hz), 6.97 (d, 2H, *J* = 8.4 Hz), 2.54 (s, 3H), 2.45 (appt, 2H, *J* = 7.7 Hz), 1.45 (appquint, 2H, *J* = 7.6 Hz), 1.23 (appsextet, 2H, *J* = 7.4 Hz), 0.85 (t, 3H, *J* = 7.4 Hz).

#### 4.1.5. N-(4-butylphenyl)-3-[4-(2-hydroxyphenyl)piperazine-1-carbonyl]-4-methylbenzene-1-sulfonamide (TOS-1)

Briefly, 1,1′-carbonyldiimidazole (92 mg, 1.05 eq) was added portionwise to a solution of 5-[(4-butylphenyl)sulfamoyl]-2-methylbenzoic acid (188 mg, 0.54 mmol) in 0.2 mL of CH_2_Cl_2_. After the cessation of the gas evolution, the reaction mixture was stirred further for 15 minutes. Three mL of 2-propanol and 4-(2-hydroxyphenyl)piperazin-1-ium hydrochloride (139 mg, 1.2 eq) were added to the resulting compound N-(4-butylphenyl)-3-(1H-imidazole-1-carbonyl)-4-methylbenzene-1-sulfonamide (4), which was not isolated. The reaction mixture was irradiated with microwaves (100 W, 80 °C, 30 min). The chromatographic separation of the reaction mixture was performed on a column (D = 2 cm, L = 35 cm) that was filled with silica gel and eluted with a gradient mixture of CH_2_Cl_2_ and EtOAc (0 → 25%). After the evaporation of solvents, the residue was dissolved in a minimum amount of Et_2_O. The mixture was added dropwise to 10 parts of cold pentane. A white solid was precipitated. After filtration and air drying, TOS-1 was obtained (137 mg, 45%) as a white solid. ^1^H NMR ((CD_3_)_2_SO, 500 MHz): δ 10.07 (s, 1H), 9.03 (s, 1H), 7.67 (dd, 1H, *J* = 8.1, 1.9 Hz), 7.46 (d, 1H, *J* = 2.5 Hz), 7.47 (d, 1H, 8.1 Hz), 7.02 (d, 2H *J* = 8.4 Hz), 6.97 (d, 2H, *J* = 8.4 Hz), 6.89-6.85 (m, 2H), 6.81 (dd, 1H, *J* = 7.8, 1.4 Hz), 6.75 (apptd, 1H, *J* = 7.5, 1.4 Hz), 3.79 (m, 2H), 3.06 (m, 2H), 2.97 (m, 2H), 2.72 (m, 2H), 2.40 (appt, 2H, *J* = 7.7 Hz), 2.27 (s, 3H), 1.42 (appquint, 2H, *J* = 7.6 Hz), 1.19 (appsextet, 2H, *J* = 7.4 Hz), 0.81 (t, 3H, *J* = 7.4 Hz). NMR spectrum of TOS-1 is presented in Figure S3. 

#### 4.1.6. N-(4-butylphenyl)-3-{4-[2-(2-fluoroethoxy)phenyl]piperazine-1-carbonyl}-4-methylbenzene-1-sulfonamide (TOS-2)

Compound TOS-1 (127 mg, 0.25 mmol) was dissolved in 2 mL of dry DMF to which Cs_2_CO_3_ (82 mg, 0.25 mmol) was added. The reaction mixture was irradiated in a microwave reactor (120 °C, 100 W, 2 minutes) and 2-fluoroethyl 4-methylbenzene-1-sulfonate was injected. Irradiation was continued for 15 minutes. The DMF was evaporated and the reaction mixture was separated on a flash chromatographic cartridge that had been eluted with gradient mixtures of CH_2_Cl_2_ and EtOAc (0 → 25%). A waxy colorless solid (45 mg, yield %) was obtained and solidified as a white solid. An analytical sample was prepared by dissolving the wax in a minimal portion of diethyl ether and adding this solution to a stirred portion of cold pentane. ^1^H NMR ((CD_3_)_2_SO, 500 MHz): δ 10.06 (s, 1H), 7.67 (dd, 1H, *J* = 8.1, 2.0 Hz), 7.47 (d, 1H, *J* = 8.1 Hz), 7.46 (d, 1H, *J* = 1.5 Hz), 6.90-7.03 (m, 8H), 4.76 (m, 2H, *J* = 47.8 Hz), 4.24 (m, 2H, *J* = 30.3 Hz), 3.77 (m, 2H), 3.07 (m, 2H), 3.04 (m, 2H), 2.80 (m, 2H), 2.38 (appt, 2H, *J* = 7.7 Hz), 2.26 (s, 3H), 1.41 (appquint, 2H, *J* = 7.6 Hz), 1.19 (appsextet, 2H, *J* = 7.4 Hz), 0.80 (t, 3H, *J* = 7.4 Hz). NMR spectrum of TOS-2 is presented in Figure S3.

#### 4.1.7. Docking Studies

Docking to the protein kinase structures was performed using Glide XP (eXtra Precision) in the Schrodinger Suite 2019-2. The structures were prepared for docking with Protein Preparation Wizard. The conformers of the ligands were created with LigPrep and Epik. The protonation states of the proteins and ligands were calculated at pH 7.4. The protein–ligand interactions were investigated using the Protein-Ligand interaction tool in Schrodinger Maestro 12. The figures showing the 3D docking poses and interactions were also prepared with the Maestro interface.

The structural pharmacophore hypotheses were generated using an automated method (e-pharmacophores). In this approach, the hypotheses are based on the results of the docking of small molecular fragments to a receptor (Glide XP). We used two sets of fragments: the standard fragment library provided by Schrodinger and fragments containing the features from the small molecules under study. The molecules were screened against hypotheses using the Phase Ligand Screening tool in Maestro 12.

Fitness = W_RMSD_(Align Score) + w_vector_ (Vector Score) + w_volume_ (Volume Score)
Align Score = Average value of (1.0 – RMSD/1.2)
Vector Score = Average cosine between vector features
Volume Score =Average (Volume_common_/Volume_total_)

The RMSD for a given ligand-hypothesis superposition was computed from the deviations in the positions of each pair of matching pharmacophore features. 

The cosine between two vector features was determined by the smallest angle that was formed by any pair of vectors that arose from those features. Thus, when computing the vector score contribution from a pair of acceptors that were associated with carbonyl oxygens, each of which gives rise to two vectors, a total of four angles were considered. Furthermore, if a given pharmacophore contained one acceptor, one donor, and one aromatic ring, the vector score for that particular ligand–hypothesis pair was the average of the cosines from those three vector features.

The volume score was the average atom-based shape similarity between the reference conformer and the other active conformer. The shape similarities were computed from the hard-sphere overlaps between all of the heavy atoms using a single atomic radius of 1.7 Å and making no differentiation among the different types of atoms. Any pharmacophore feature overlaps were not considered at this point because the agreement in the locations and orientations of the pharmacophore features was already accounted for by the RMSD and vector scores. The volume scores emphasize the agreement in the overall generic shape of the ligands.

### 4.2. Biological Studies

#### 4.2.1. Cell Lines and Cell Culture Conditions

The human colon carcinoma cell line HCT 116 wild type (p53^+/+^) and the human breast carcinoma cell line MCF-7 were obtained from ATCC. The human colon cancer cell line HCT 116, which has a p53 deletion (p53^-/-^), was kindly provided by prof. M. Rusin from the Maria Sklodowska-Curie Memorial Cancer Centre and Institute of Oncology in Gliwice, Poland. The glioblastoma cell line U-251 was kindly provided by prof. G. Kramer-Marek from The Institute of Cancer Research in London, United Kingdom. The pancreatic cell line PANC-1 and the human chronic myelogenous leukemia cell line K562 were purchased from Sigma-Aldrich. The adherent cell lines were cultured in Dulbecco’s modified Eagle’s medium (DMEM) containing 12% heat-inactivated fetal bovine serum (all from Sigma-Aldrich) with a mix of two standard antibiotics: 1% *v*/*v* of penicillin and streptomycin (Gibco) in 75 cm^2^ flasks (Nunc). The suspension cell line K562 was cultured in an RPMI-1640 medium (Sigma-Aldrich), which had been supplemented with 10% heat-inactivated foetal bovine serum and the antibiotics. All of these cell lines were grown under standard conditions at 37 °C in a 5% CO_2_ humidified atmosphere. Prior to beginning the experiments, all of the cell lines were subjected to routine mycoplasma testing with specific *Mycoplasma* primers using the PCR technique.

#### 4.2.2. Cytotoxicity Studies

The exponentially growing cells for all of the cell lines were seeded in 96-well plates (Nunc) at a density of 5000 cells/well and incubated at 37 °C for 24 h. The assay was performed following a 72-h incubation with the various concentrations of the compounds that were being tested. Then, 20 µL of CellTiter 96^®^AQueous One Solution-MTS (Promega) solution was added to each well (with 100 µL DMEM without phenol red) and incubated for 1 h at 37 °C. The optical densities of the samples were analyzed at 490 nm using a multi-plate reader (Synergy 4, BioTek). The results are expressed as the percentage of the control and were calculated as the inhibitory concentration (IC_50_) values using GraphPad Prism 7. Each individual compound was tested in triplicate in a single experiment with each experiment being repeated five times.

#### 4.2.3. Tyrosine Kinase Inhibition in vitro

The inhibition of the tyrosine kinases by the tested compounds was measured using the Kinase Selectivity TK-2 Profiling Systems (Promega) as was previously reported [68,69]. Briefly, the kinases (ABL1; BRK; BTK; CSK; Fyn A; Lck; Lyn B; Src) and substrate/co factor strips were prepared according to the manufacturer’s instructions. The compounds being tested were dissolved in DMSO to a concentration of 100 μM, which was then used as the stock solution to prepare the 0.5 μM final concentration in a 1x Kinase Reaction Buffer (40 mM Tris – pH 7.5; 20 mM MgCl_2_; 0.1 mg/mL BSA; 50 μM DTT). The experiment began with transferring 1 μl of the prepared solutions of the tested compounds onto a 384-well white plate that was designed for luminescence (Corning). Then, 2 μL of the kinases from the eight-well strip were added into each well and the plate was incubated for 10 min at room temperature (RT), after which 2 μL of the substrates from the eight-well substrate/co-factor strip were added to each well and the plate was left at RT for 1 h. Solutions of 1x Kinase Reaction Buffer with 5% vehicle (DMSO) were used as the negative controls without an inhibitor or enzymes. The activity of each kinase was determined using an ADP-Glo Kinase Assay (Promega). Firstly, in order to stop the reaction, 5 μL of ADP-Glo reagent was added to each well and the plate was incubated for 40 min at RT. Afterwards, 10 μL of the Kinase Detection Reagent was added to each well and the luminescence was measured in a Synergy 4 multi-plate reader following a 30-min incubation at RT. The experiments were performed at least three times. The data are expressed as the percentage of the inhibition of tyrosine kinase after treatment with the tested compounds. Additionally, the IC_50_ values were calculated from three independent experiments with various concentrations of TOS-1, TOS-2 and axitinib using GraphPad Prism 7.

#### 4.2.4. ABL1 T315I Kinase Assay

The inhibition of the ABL1 mutant form by TOS-1, TOS-2 and axitinib was measured using an ABL (T315I) Kinase Assay (Promega) according to the supplier’s protocols. The commercially available kit contains the mutant ABL1 enzyme ready for testing. Briefly, the solutions of the compounds being tested at various concentrations were prepared in a 1x Kinase Reaction Buffer. Firstly, 1 μl of the solutions of the tested compounds or vehicle – 5% DMSO (as the control without an inhibitor) were added onto a 384-well low volume white plate. Then, 2 μL of the ABL1 T315I enzyme was added into each well. After 10 min of incubation at RT, 2 μL of the substrate/ATP mixture was added to each well and incubated for 1h at RT. The luminescence of the kinase activity after treatment with the tested compounds was measured using an ADP-Glo Kinase Assay as was described above. The experiments were performed three times and the results are presented as the IC_50_ values, which were calculated using GraphPad Prism 7.

#### 4.2.5. Pharmacological Synergy on the ABL Enzyme Model

The inhibition of the ABL enzyme by the combination of TOS inhibitors and axitinib were measured using an ABL1 Kinase Assay (Promega) according to a methodology that was similar to the one described above. The experiments were performed at least three times. The synergy was calculated according to the Chou-Talay method (CompuSyn software) [33,70]. Briefly, the concentration range was adjusted to cover the specified IC_50_ value against the ABL1 enzyme for each inhibitor. Five concentrations of the TOS inhibitors and axitinib were tested. The doses were increased at a constant ratio according to refs [33,70] (Table S1). 

The combination index CI was calculated according to equation

$$\frac{{\left(D\right)}_{TOS}}{{\left(D_{x}\right)}_{TOS}}+\frac{{\left(D\right)}_{axitinib}}{{\left(D_{x}\right)}_{axitinib}}=CI$$

where D_x_ was the dose of TOS or axitinib given alone that was enough to achieve effect x (x% of the inhibition of the enzyme activity). D was the portion of TOS or axitinib given in a combination that was sufficient to achieve effect x. 

The fraction affected f_a_ was calculated according to equation

$$f_{a}={\left(\frac{D}{D_{m}}\right)}^{m}/f_{u}$$

where D was the dose of the TOS or axitinib, D_m_ jest was the dose of the TOS or axitinib that caused a 50% effect, m described the shape of the dose-response curve, and f_u_ was the unaffected fraction. 

#### 4.2.6. Mutant IDH1 Activity – D-2-hydroxyglutarate Production

The K562 and U-251 cells were seeded in 3-cm Petri dishes (Nunc) at a density of 0.5 × 10^6^ cells/well and incubated at 37 °C for 24 h. Then, the medium was removed and freshly prepared solutions of the tested compounds: TOS-1 (30 μM and 50 μM), TOS-2 (30 μM and 50 μM) and axitinib (0.5 μM and 50 μM) were added for the leukemia and glioblastoma cells, respectively. After a 24-h treatment, the assays were performed using a D-2-Hydroxyglutarate Assay Kit (Abcam) according to the manufacturer’s instructions. Briefly, 25 μL of each cell lysate was transferred onto 96-well plates. Internal and external standards (containing 1 mM D2HG) were also prepared. Then, a freshly prepared reaction mix containing D2HG Assay Buffer, D2HG Enzyme and D2HG Substrate Mix was added to each well. Reaction mixes with the cell lysates were incubated for 60 min at 37 °C in the dark. The optical densities of the samples were analyzed at 450 nm using a Synergy 4 multi-plate reader. The experiments were performed at least four times. The D-2-Hydroxyglutarate concentration is expressed as nM/μL.

#### 4.2.7. Analysis of the mRNA Expression of the *IDH1* and Non-Receptor Tyrosine Kinases

The total RNA was isolated from the all of the cell lines using a TRIzol Reagent (Ambion) according to the manufacturer’s instructions. Single-stranded cDNA was synthetized from 5 μg of total RNA using a SuperScript™ IV Reverse Transcriptase kit (Invitrogen) and Oligo(dT)_20_ Primers (Invitrogen). Quantitative Real-Time PCR was performed using a CTX96 Touch™ Real-Time PCR Detection System (Biorad) in a 20 μL reaction volume. The PCR reaction consisted of SsoAdvanced™ Universal SYBR^®^ Green Supermix (Biorad), a primer pair mix for each target (0.5 μM each) and 1 μL of cDNA. All of the primer pair sequences were designed in Primer 3 and were purchased from Sigma-Aldrich (Table S2). The PCR reactions were performed under the following conditions: initial denaturation at 95 °C for 30 sec followed by 40 denaturation cycles at 95 °C, 15 sec, annealing (primer-specific temperature for 30 sec) and extension at 72 °C for 60 sec. The data obtained from the qRT-PCR experiments were analyzed based on the normalized expression of the target genes to a reference gene – *GAPDH*, and then compared to a calibrator sample using the 2^-ΔΔCT^ method. The experiments were performed at least four times. 

#### 4.2.8. Statistical Analysis

The results are expressed as the mean ± standard deviation (SD) from at least three independent experiments. Statistical analysis for the 2-HG measurements were performed using a one-way ANOVA with a Bonferroni post-hoc test using GraphPad Prism 7 software. A p-value of 0.05 or less was considered to be statistically significant. The CI indexes with error values were calculated using CompuSyn software.

## 5. Conclusions

Tosylamides, which were designed as selective IDH1 R132H inhibitors, also had an interesting activity against the non-receptor tyrosine kinases, particularly ABL1. Both tested compounds were effective in blocking ABL1 kinase and lowering the 2-hydroxyglutarate in cancer cells. Moreover, their dual inhibitory potency helped to explain the differences in their anticancer activity towards a of cancer cell lines. Further investigation suggested an allosteric inhibition of ABL1 *via* interactions with the myristate binding pocket. This was confirmed by the synergistic interactions in combination with axitinib and the docking studies. Pharmacophore models for the specific binding sites of the ABL1 and IDH1 enzymes were built and compared. Our results showed that the common features between these models can help to explain their overall potency via their multi-targeted activity. Moreover, tosylamides can be regarded as interesting leading structures for novel multifunctional ABL1-IDH1 inhibitors. For the first time inhibitors of cytosolic kinases has been connected with activity on proto-oncogenic mutation of isocitrate dehydrogenase. Mutual regulatory effect exerted on cell’s survival and proliferation by these proteins demonstrate their promising value as targets in anticancer therapy. The molecular flexibility of TOS may support the design of novel multi-targeted inhibitors.

## 6. Associated Content

The Supporting Information contains a table with the Fa fractions that resulted from the applied doses of the TOS inhibitors and axitinib in pharmacological synergy on the ABL enzyme model and a list of primer pair sequences that were used to determine the mRNA expression. Spectra for chemical characterization of the compounds synthesized in this study are also presented. 

## Figures and Tables

**Table 1:** Inhibition of the activity of the kinases after treatment with the IDH1 inhibitors axitinib and imatinib that were tested at a concentration of 0.5 μM.

Compound No.	Inhibitory Effect of the Non-Receptor Tyrosine Kinase Activity [%]								
	ABL1 native	ABL1 T315I	BRK	BTK	CSK	Fyn A	Lck	Lyn B	Src
**TOS-1**	42.82 ± 8.46	29.34 ± 7.43	22.65 ± 0.94	32.26 ± 7.43	38.44 ± 6.15	29.93 ± 4.29	34.73 ± 2.51	0	0
**TOS-2**	59.24 ± 8.12	26.13 ± 6.61	0	50.87 ± 1.87	9.66 ± 3.59	19.65 ± 2.78	0	0	13.75 ± 4.23
**Axitinib**	56.63 ± 9.37	77.84 ± 9.10	0	0	11.37 ± 3.89	43.81 ± 2.96	10.08 ± 3.83	38.96 ± 9.95	10.80 ± 1.95
**Imatinib**	77.17 ± 3.85	0	0	0	0	0	0	0	0

**Table 2:** The antiproliferative activity of the tested IDH1 inhibitors compared to axitinib and imatinib in several tumor cell lines.

Compound No.	IC_50_ values [μM]					
	K562	U-251	HCT 116 p53^+/+^	HCT 116 p53^-/-^	MCF-7	PANC-1
**TOS-1**	12.65 ± 3.20	27.59 ± 0.17	24.69 ± 0.32	27.83 ± 0.17	18.09 ± 1.00	25.25 ± 3.38
**TOS-2**	17.26 ± 2.23	>30	24.44 ± 0.82	28.85 ± 1.07	15.59 ± 1.35	18.93 ± 0.42
**Axitinib**	0.10 ± 0.01	>30	>30	>30	>30	>30
**Imatinib**	0.13 ± 0.03	>30	>30	>30	>30	>30

## Supplementary Materials

The following are available online at https://www.mdpi.com/2072-6694/12/3/536/s1, Figure S1: Comparison of hypotheses. Figure S2: Docking poses of inhibitors. a (GNF-2/ABL1), b (TOS-2/ABL1) c (TOS2/IDH1) in pharmacophore forced poses docked into enzyme pockets, d (GNF-2/ABL1), e (TOS-2/ABL1) f (TOS2/IDH1) ligand docked directly to protein. Figure S3: ^1^H NMR of TOS-1 and TOS-2 ligands. Table S1: Fa fractions resulted from the respective TOS inhibitors or axitinib doses. Table S2: Primer pair sequences that were used to determine the mRNA expression of the *IDH1*, non-receptor tyrosine kinases and *GAPDH*. Table S3: List of input fragments for hypothesis.

## Abbreviations

2-HG - D-2-hydroxyglutarate, CI - combination index, DMSO - dimethyl sulfoxide, DTT - ditiotreitol, GBM - glioblastoma, HIF - hypoxia-inducible factor, IDH1 - isocitrate dehydrogenase 1, JmjC - Jumonji C domain, KDM - histone lysine (K)-specific demethylase, MMPs - matrix metalloproteinases, NADP^+^ - nicotinamide adenine dinucleotide phosphate, NADPH - reduced nicotinamide adenine dinucleotide phosphate, PDGF - platelet-derived growth factor, qRT-PCR - Quantitative Real-Time PCR, TCA - tricarboxylic acid cycle, TET - ten-eleven translocation methylcytosine dioxygenase, TGF-β - transforming growth factor β, VEGF - vascular endothelial growth factor, VEGFR2 - vascular endothelial growth factor receptor 2, α-KG - α- ketoglutarate.
