# Supplementary material for: The Landscape of the Anti-Kinase Activity of the IDH1 Inhibitors
Source: Cancers (Basel). 2020 Feb 26;12(3):536. doi: 10.3390/cancers12030536 (PMC7139656; doi:10.3390/cancers12030536)
Supplement: Supplementary file 1 [file cancers-12-00536-s001.pdf]

The Landscape of the Anti-Kinase Activity of the IDH1 Inhibitors

Katarzyna Malarz [1,#,*], Jacek Mularski [2,#], Marcin Pacholczyk [3] and Robert Musiol [2,*]

[1] August Chełkowski Institute of Physics and Silesian Center for Education and Interdisciplinary Research, University of Silesia in Katowice, 75 Pułku Piechoty 1, 41-500 Chorzów, Poland
[2] Institute of Chemistry, University of Silesia in Katowice, 75 Pułku Piechoty 1A, 41-500 Chorzów, Poland; jacek.mularski@gmail.com
[3] Silesian University of Technology, Department of Systems Biology and Engineering, Akademicka 16, 44-100 Gliwice, Poland; marcin.pacholczyk@polsl.pl

[#] These authors contributed equally
* Correspondence: katarzyna.malarz@us.edu.pl (K.M.); robert.musiol@us.edu.pl (R.M.)

Table of contents:

Table S1. Fa fractions resulted from the respective TOS inhibitors or axitinib doses.

	axitinib				
TOS-1	dose [µM]	0.125	0.25	0.5	1
dose [µM]	F_a	0.344349	0.407227	0.501202	0.585215
0.375	0.112572	0.378733			
0.625	0.414853		0.483741		
1.25	0.559027			0.720406	
2.5	0.586297				0.841235

	axitinib					
TOS-2	dose [µM]	0.0625	0.125	0.25	0.5	1
dose [µM]	F_a	0.213479	0.344349	0.407227	0.501202	0.585215
0.0625	0.072449	0.353985				
0.125	0.247466		0.463196			
0.25	0.373724			0.621545		
0.5	0.517996				0.68516	
1	0.638019					0.810094

Table S2. Primer pair sequences that were used to determine the mRNA expression of the *IDH1*, non-receptor tyrosine kinases and *GAPDH*.

Gene	GenBank accession no.	Forward primer (5′→3′)<br>Reverse primer (5′→3′)
IDH1	NM_001282387	CAGGCTCATAGATGACATGGTGG<br>CACTGGTCATCATGCCAAGGGA
ABL1	NM_005157	GAAGCCGCTCGTTGGAACT<br>CCTAAGACCCGGAGCTTTTCA
BTK	NM_001287345	ACAGATTCCGAGGAGAGGTGAGG<br>GGTCCTTCATCATATACAACCTGGAATGG
BRK	NM_001256358	ATGAAGAAGCTGCGGCACAA<br>CCGAAACGGGCAGGACTT
CSK	NM_001127190	GGCTCTACATCGTCACTGAG<br>CTCAGACACCAGCACATTG
FYN	NM_002037.5	GTAGTCATGGCAACCCGCTA<br>ACAACCCCCACCCTCATTTC
LCK	NM_001042771	AGCTTTTCTGTGGCTGGTGA<br>CATTTCGGATGAGCAGCGTG
LYN	NM_001111097	GCGAGCGGGAAATATGGGAT<br>AGATTCTGGAACTGGTTGAGTCT
SRC	NM_005417	CCTCGTGCGAGAAAGTGAG<br>GGCGTTGTCGAAGTCAG
GAPDH	NM_002046	GAGTCAACGGATTTGGTCGTA<br>GCCCCACTTGATTTTGGAG

Figure S1. Comparison of hypotheses.

GNF-2 and GNF-5 matched to hypothesis generated in regard to standard fragments library (left) and on the basis of fragments listed in Table S3. Both hypotheses display similar level of matching to ring features. Hypothesis based on our library reveals hydrophobic feature to which trifluoromethyl moiety fits. Standard library display acceptor feature to which approaches oxygen of amide moiety. This show that hypotheses were sensitive to initial conditions, which was different set of probes.

Standard library customized

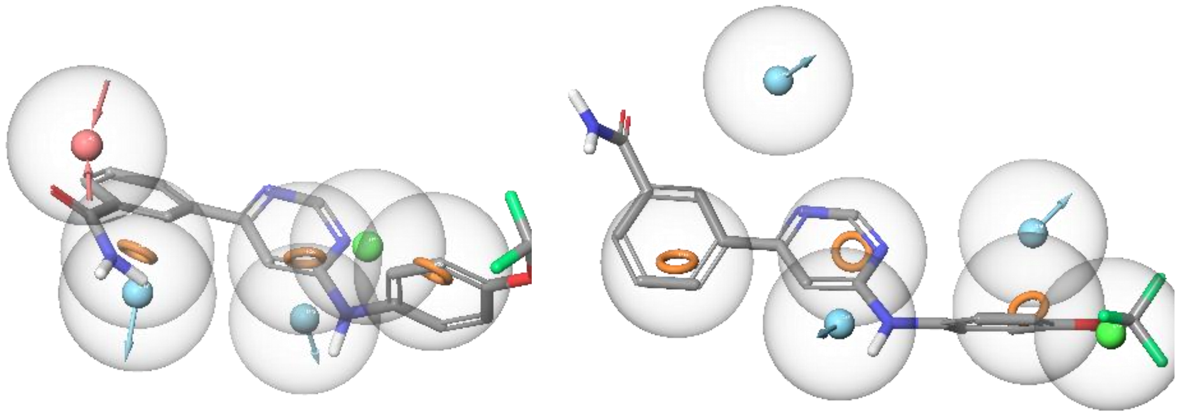

|-------------------GNF-2-------------------|

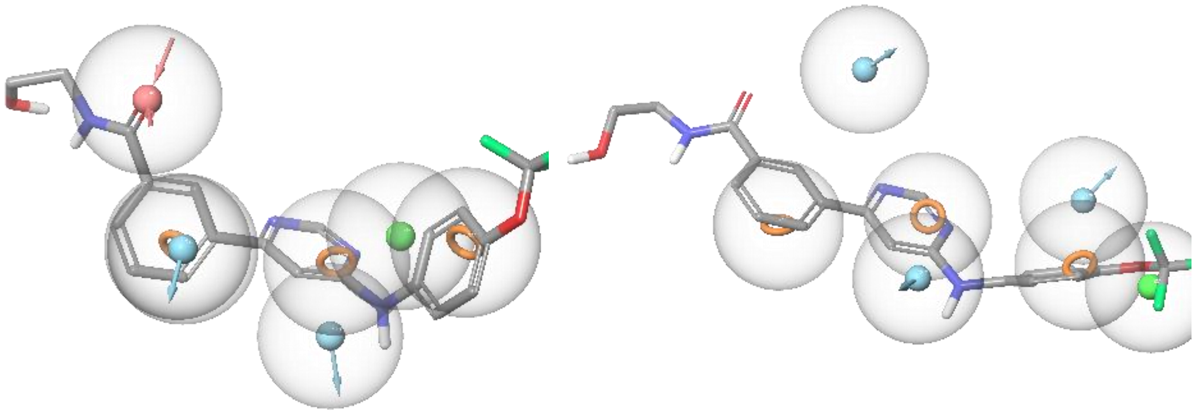

|-------------------GNF-5-------------------|

Table S3. List of input fragments for hypothesis.

c1ccccc1
Cc1ccc(cc1)S(=O)(C)=O
Cc1ccc(cc1)S(=O)(N)=O
Cc1ccc(cc1)S(=O)(=O)NC
Cc1ccc(cc1)S(=O)(=O)Nc2ccccc2
O=S(C)(=O)Nc1ccccc1
O=S(C)(=O)Nc1ccc(C)cc1
CCCCc1ccccc1
Cc1ccccc1
Cc1ccccc1C(=O)NC
Cc1ccc(cc1C(=O)NC)S(=O)(=O)NC
CN1CCN(C)CC1
O=C(N1CCN(C)CC1)c2ccccc2C
CN1CCN(CC1)c2ccccc2
CN1CCN(CC1)c2ccccc2OC
FCCOC
CN1CCN(CC1)c2ccccc2OCCF
FCCOc1ccccc1
CNc1ccccc1OCCF
COc1ccccc1
COc1ccccc1NC
C/C(NC)=C(\C)OC
CCS(=O)(=O)NC
CCC(=O)NC
O=C(CC)N1CCN(C)CC1
O=C(CC)N1CCN(CC1)c2ccccc2
O=C(CC)N1CCN(CC1)c2ccccc2OC
O=C(CC)N1CCN(CC1)c2ccccc2OCCF
O=S(C)(=O)Nc1ccc(cc1)CCCC

Figure S2. Docking poses of inhibitors. a (GNF-2/ABL1), b (TOS-2/ABL1) c (TOS2/IDH1) in pharmacophore forced poses docked into enzyme pockets, d (GNF-2/ABL1), e (TOS-2/ABL1) f (TOS2/IDH1) ligand docked directly to protein.

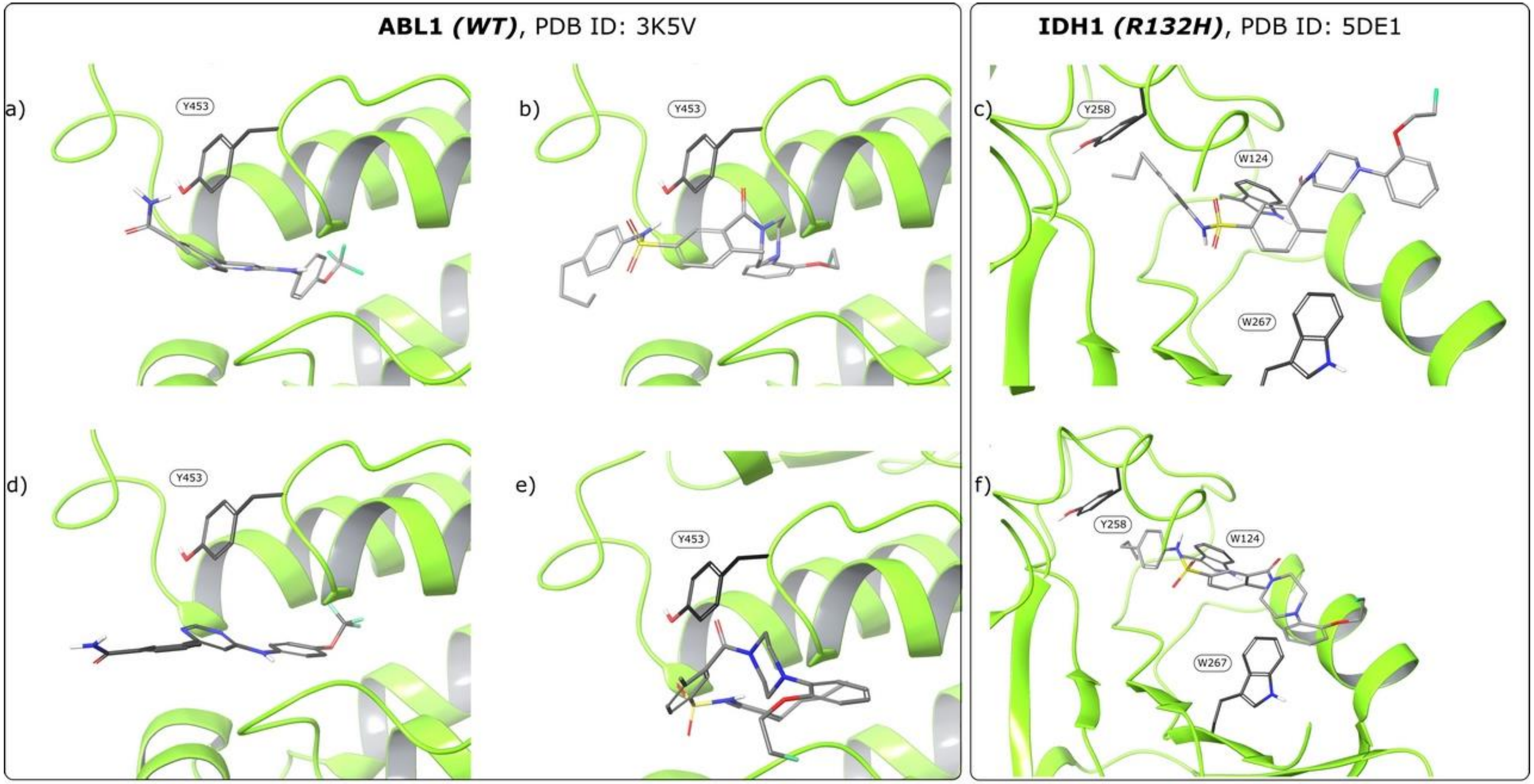

Figure S3. ¹H NMR of TOS-1 and TOS-2 ligands.

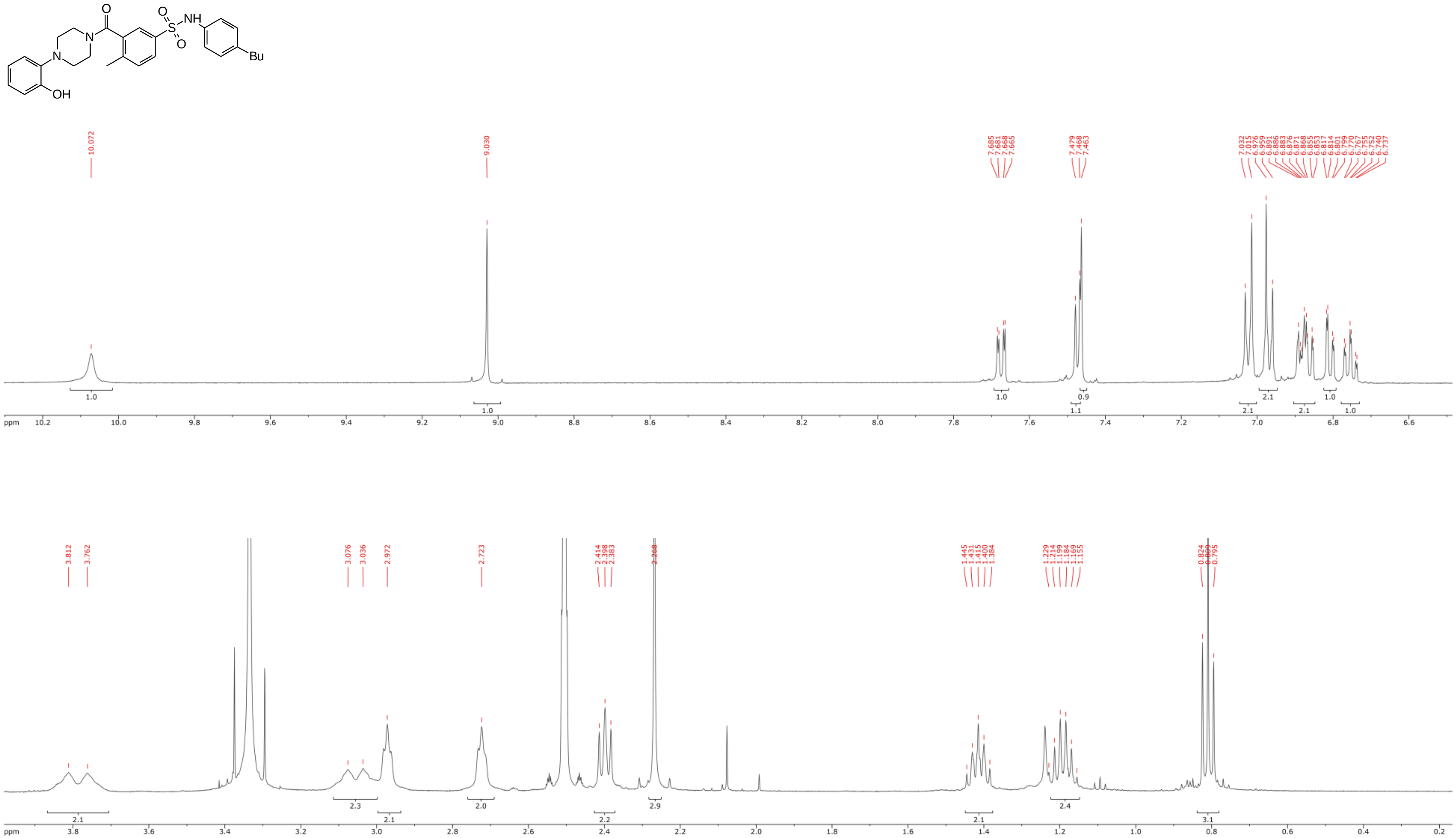

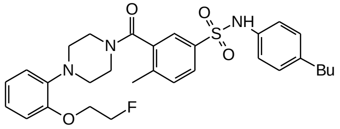

O
N
N
O
F
O
S
O
NH
Bu

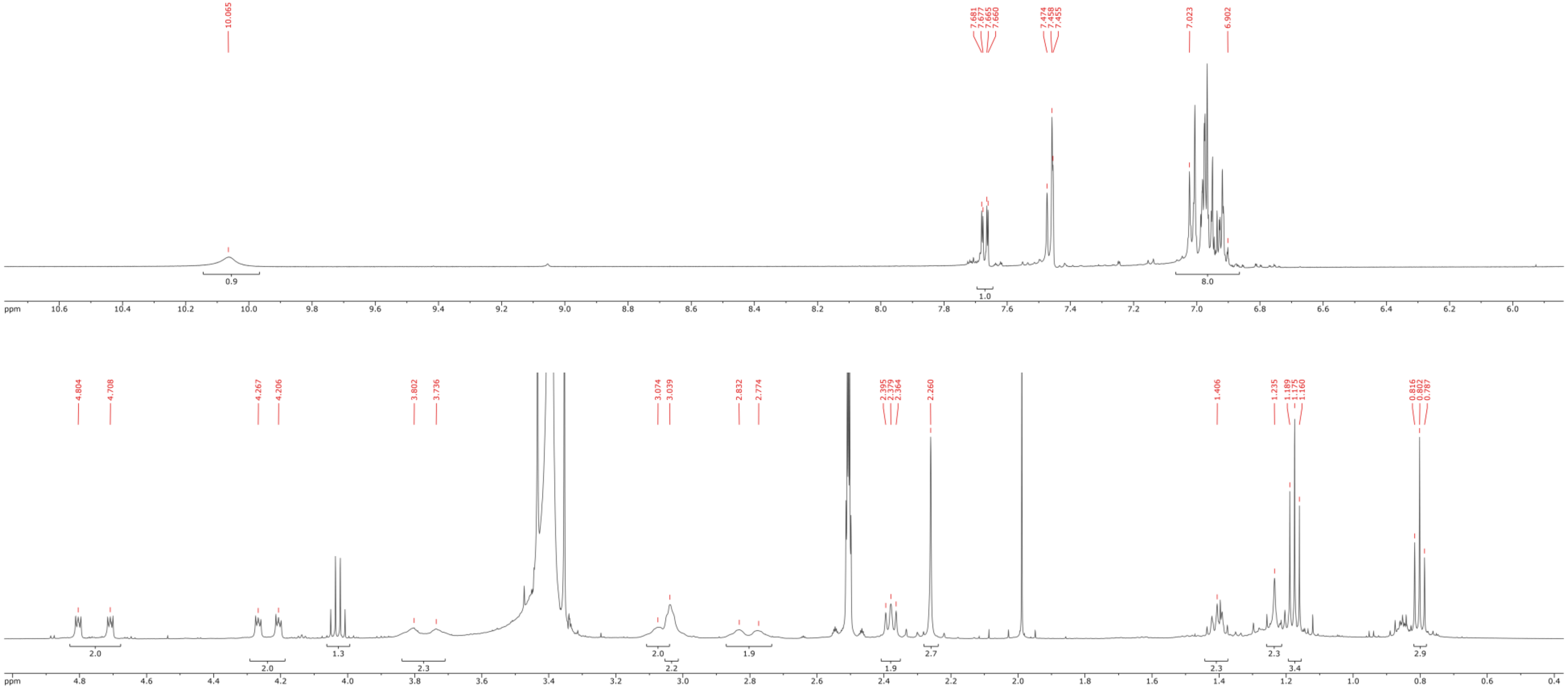

10.065
7.681
7.677
7.665
7.660
7.474
7.458
7.455
7.023
6.902
0.9
1.0
8.0
ppm
10.6
10.4
10.2
10.0
9.8
9.6
9.4
9.2
9.0
8.8
8.6
8.4
8.2
8.0
7.8
7.6
7.4
7.2
7.0
6.8
6.6
6.4
6.2
6.0
4.804
4.708
4.267
4.206
3.802
3.736
3.074
3.039
2.832
2.774
2.395
2.379
2.364
2.260
1.406
1.235
1.189
1.175
1.160
0.816
0.802
0.787
2.0
2.0
1.3
2.3
2.0
2.2
1.9
1.9
2.7
2.3
2.3
3.4
2.9
ppm
4.8
4.6
4.4
4.2
4.0
3.8
3.6
3.4
3.2
3.0
2.8
2.6
2.4
2.2
2.0
1.8
1.6
1.4
1.2
1.0
0.8
0.6
0.4
